# Pathological alterations in the gastrointestinal tract of a porcine model of DMD

**DOI:** 10.1186/s13578-021-00647-9

**Published:** 2021-07-15

**Authors:** Xiaodong Zou, Hongsheng Ouyang, Daxin Pang, Renzhi Han, Xiaochun Tang

**Affiliations:** 1 Jilin Provincial Key Laboratory of Animal Embryo Engineering, College of Animal Sciences, Jilin University, Changchun, Jilin People’s Republic of China; 2 Department of Surgery, Davis Heart and Lung Research Institute, Biomedical Sciences Graduate Program, Biophysics Graduate Program, The Ohio State University Wexner Medical Center, Columbus, OH 43210 USA

**Keywords:** CRISPR, Duchenne muscular dystrophy, Genome editing, Gastrointestinal tract, Pig, Porcine, Swine

## Abstract

**Background:**

Patients with Duchenne muscular dystrophy (DMD) develop severe skeletal and cardiac muscle pathologies, which result in premature death. Therefore, the current therapeutic efforts are mainly targeted to correct dystrophin expression in skeletal muscle and heart. However, it was reported that DMD patients may also exhibit gastrointestinal and nutritional problems. How the pathological alterations in gastrointestinal tissues contribute to the disease are not fully explored.

**Results:**

Here we employed the CRISPR/Cas9 system combined with somatic nuclear transfer technology (SCNT) to establish a porcine model of DMD and explored their pathological alterations. We found that genetic disruption of dystrophin expression led to morphological gastrointestinal tract alterations, weakened the gastrointestinal tract digestion and absorption capacity, and eventually led to malnutrition and gastric dysfunction in the DMD pigs.

**Conclusions:**

This work provides important insights into the pathogenesis of DMD and highlights the need to consider the gastrointestinal dysfunction as an additional therapeutic target for DMD patients.

**Supplementary Information:**

The online version contains supplementary material available at 10.1186/s13578-021-00647-9.

## Introduction

Duchenne muscular dystrophy (DMD), inherited in an X-linked recessive manner, is a severe and progressive neuromuscular disorder [1], and its prevalence in the general population is approximately 1/5000 [2, 3]. Progressive muscle injury and degeneration in DMD patients leads to muscular weakness, loss of ambulation, respiratory impairment, and cardiomyopathy [4]. Although the clinical and pathological progression of skeletal muscle and myocardium involvement can be variable, death usually occurs around the age of 30 years due to cardiac and/or respiratory failure [5].

DMD is caused by mutations in the *DMD* gene located at Xp21, which codes for the dystrophin protein [6], a cytoskeletal protein that functions in the muscle force transmission and sarcolemmal stability of muscle fibers [7]. With the advancing pathological progression of DMD, patients often suffer from gastrointestinal (GI) complications [8]. In DMD, reduced food intake was associated with oral muscle weakness and dysphagia, swallowing disorders, reduced chewing, prolonged mealtime, and respiratory muscle weakness [9]. Previous clinical studies reported that impaired GI smooth muscle functions cause acute gastric dilatation in DMD patients. The plain abdominal radiograph showed a dilated stomach and electrogastrogram revealed marked bradygastria [10]. DMD patients were also reported to develop the life-threatening acute gastroparesis [11]. Moreover, smooth muscle fibrosis was found in the entire GI tract of the DMD patients with intestinal pseudo-obstruction, and it was most obvious in the esophagus and stomach [12].

In the *mdx* mouse, the most commonly used animal model of DMD, the lack of dystrophin causes decrease in colonic smooth muscle contractility, peristalsis, and GI transit [13]. Compared with wild-type, *mdx* mice exhibited thickening of colonic smooth muscle layers, delayed stress-induced defecation and decreased expression of neuronal nitric oxide synthase [14, 15]. The thickness of the ileum muscle layer of *mdx* mice was reduced by 27%, the mucosal layer was partially damaged, and the mitochondria of the intestinal muscle cells was partially damaged [16]. In addition, the transport speed of the small intestine was also delayed in *mdx* mice [17]. Furthermore, the *mdx* mice have a higher concentration of collagen fibers in the submucosal region of the GI tract than wild-type (WT) mice. These alterations are presumably consequences of the loss of dystrophin protein [17–19].

As pigs share a high degree of similarity with the physiological and anatomical characteristics of humans compared to mice [20]. Like humans, pigs are true omnivores, and their balanced nutritional requirements are particularly similar to the dietary requirements of humans [21]. Of the available animal models, pigs exhibit the closest time required for intestinal transformation and digestive efficiency to those in humans [22].

Therefore, understanding the functional and morphological alterations of the GI tract in the DMD pig model could have important implications for therapeutic interventions. In this study, we used the CRISPR/Cas9 technology to create a porcine model of DMD by targeting *DMD* exon 51 and reported the pathological alterations in the GI tract of these animals.

## Results

### Design of the *DMD* gene targeting strategy

The dystrophin protein shares a high homology among species. Amino acid sequence alignment results revealed that the porcine dystrophin protein is highly homologous to the human dystrophin protein with 94.16% homology (Additional file 1: Fig. S1A). We designed a single guide RNA (sgRNA) targeting pig *DMD* exon 51 (Additional file 1: Fig. S1B). The on-target editing efficiency of this sgRNA was assessed by Sanger sequencing of the target site PCR amplicon following electroporation into porcine fetal fibroblasts (PFFs). Multipeaks around the Cas9 cleavage site were observed, indicating that this sgRNA is effective in targeting *DMD* exon 51 (Additional file 1: Fig. S1C). To assess the off-target activity, we selected the top eight potential off-target sites for this sgRNA identified by in silico analysis (Additional file 1: Table S1), and sequenced the PCR amplicons for all these eight sites with the primers listed in Additional file 1: Table S2. No overlapping peaks were found in the sequencing traces, suggesting that the editing activities at these potential off-target sites remain undetectable for the sensitivity of this assay (Additional file 1: Fig. S1D).

### PFFs with engineered mutations in *DMD* exon 51 showed impaired cell membrane integrity and early apoptosis

**Fig. 1:**
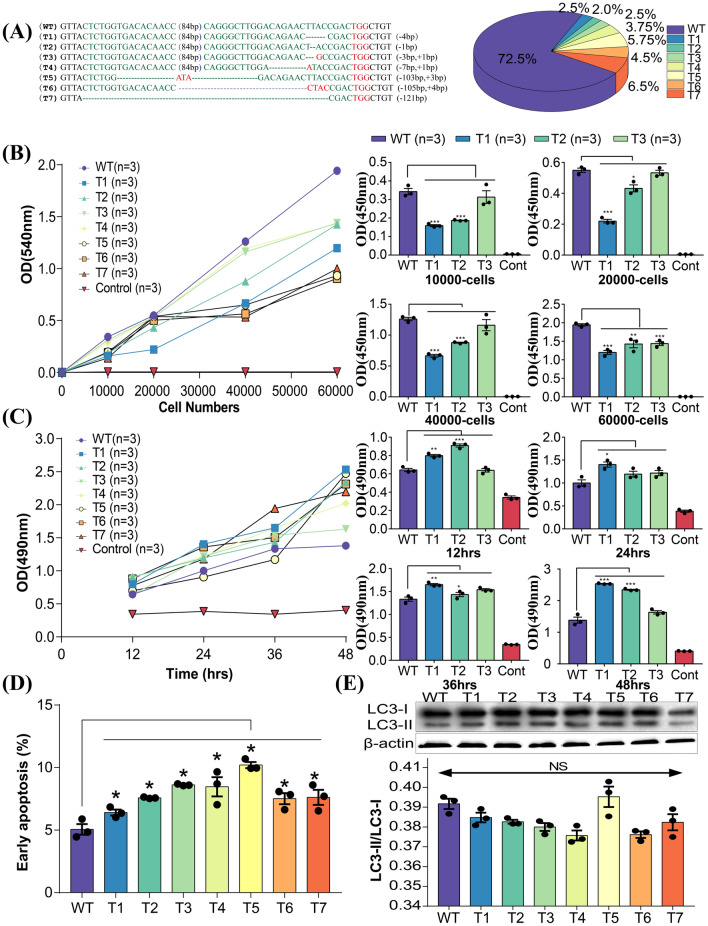
PFFs with *DMD* exon 51 deficiency showed impaired cell membrane integrity and early cell apoptosis. **A** Sanger sequencing of PFFs showed different mutations induced by Cas9/sgRNA electrotransfection. WT sequence is shown at the top of the targeting sequence. PAM sequences are highlighted in red. **B** NRD uptake assay of PFF clones carrying *DMD* exon 51 mutations at different cell densities. ***P < 0.001, **P < 0.01 and *P < 0.05. **C** LDH activities in culture medium at different time points were measured by the LDH-kit. ***P < 0.001, **P < 0.01 and *P < 0.05. **D** Cell apoptosis was analyzed by flow cytometry. *P < 0.05. **E** Western blotting analysis of autophagy in PFFs did not detect significant difference between WT and *DMD*-mutant PFFs

PFFs with *DMD* exon 51 disruption following electroporation were chosen as the donor cells for somatic nuclear transfer. A total of 400 individual PFF cell clones were analyzed and 113 (27.5%) were found to carry seven different types of mutations (Fig. 1A, Additional file 1: Table S3). All mutation types of PFFs showed abnormalities in the mRNA and protein levels of the *DMD* gene (Additional file 1: Fig. S2A, B).

Previous research showed that dystrophin protein functions to stabilize the integrity of the muscle fiber membrane. To examine whether the mutations in PFFs induced by the CRISPR/Cas9 system disrupt cell membrane integrity, we performed neutral red dye (NRD) uptake and lactate dehydrogenase (LDH) release assays. As shown in Fig. 1B and Additional file 1: Fig. S2C, the NRD uptake of PFFs with mutations in *DMD* exon 51 was significantly lower than that of PFFs from the control group, suggesting that the *DMD* mutant PFFs have lower viability. Consistent with the results of the NRD uptake assay, the LDH activity was remarkably elevated in the culture medium of PFFs with *DMD* exon 51 mutations compared to that in the culture medium of control PFFs (Fig. 1C, Additional file 1: Fig. S2D). Increasing evidence has shown that apoptosis and autophagy may play important roles in DMD [23, 24]. Therefore, we compared apoptosis and autophagy between gene-edited and WT PFFs. As shown in Fig. 1D and Additional file 1: Fig. S2E, F, increased early apoptosis in PFFs carrying mutations in *DMD* exon 51 was observed as compared to control PFFs. By assaying the conversion of LC3B-I to LC3B-II, we found no evidence of increased autophagy in gene-edited PFFs (Fig. 1E). Finally, we found no significant differences in blastocyst development rate between the control and gene-edited PFFs (Additional file 1: Fig. S2G, H; Table S4). These results demonstrated that *DMD* exon 51 disruption led to a compromised cell membrane integrity and caused early apoptosis but did not affect the developmental potential of PFFs.

### Generation of Bama miniature pigs with *DMD* exon 51 mutations

**Fig. 2:**
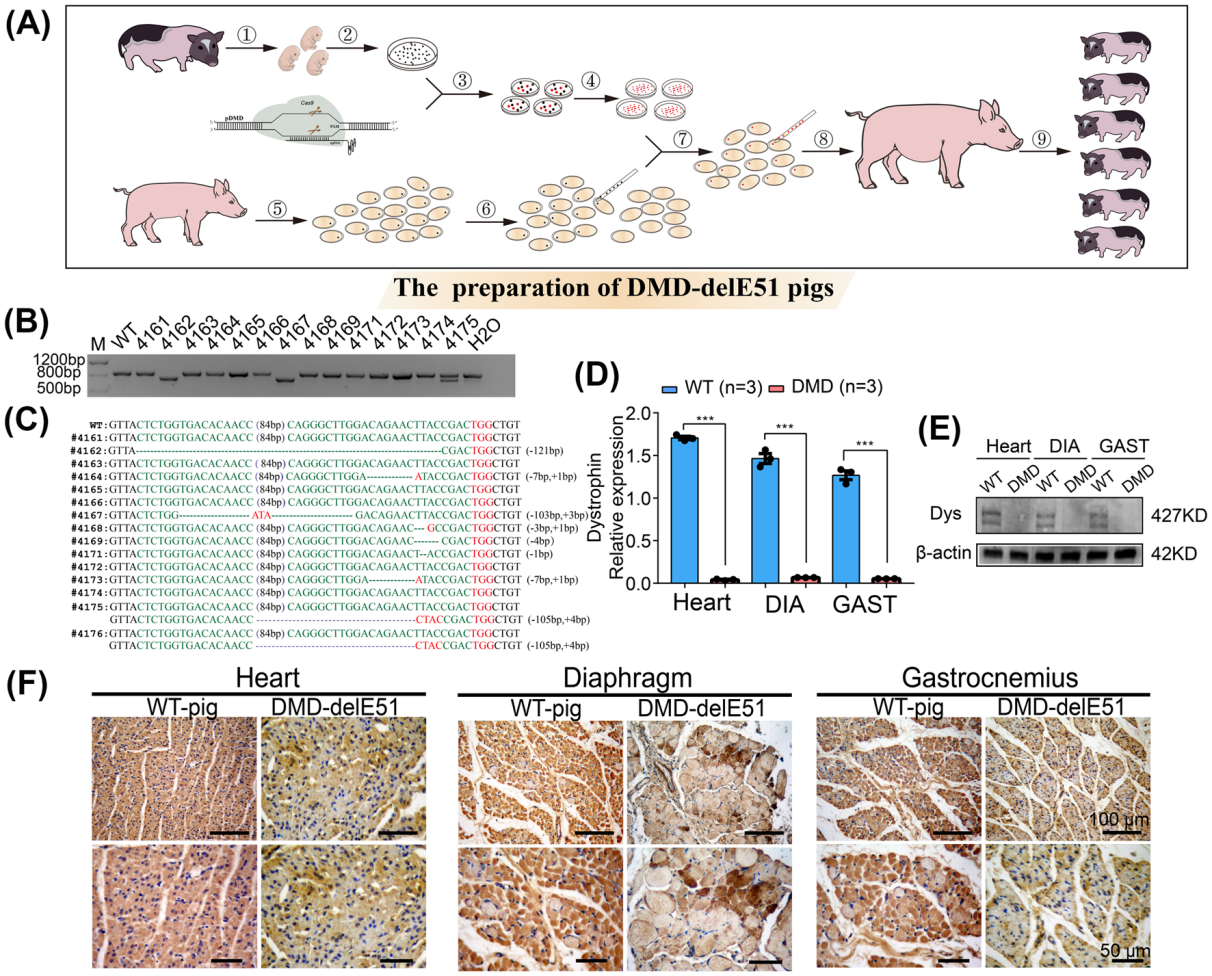
Generation and identification of *DMD* exon 51 defective pigs. **A** The construction flowchart of Bama miniature pigs with edited *DMD* exon 51. ①② Isolation and culture of miniature PFFs; ③ Transfection of miniature PFFs with Cas9/sgRNA; ④ Single cell clone picking and culturing; ⑤ Acquisition of oocytes; ⑥ Enucleation of oocytes; ⑦ Somatic cell nuclear transfer; ⑧ Embryo transfer; ⑨ Delivery and identification of DMD-delE51 pigs. **B** PCR analysis of *DMD* exon 51 in all piglets. **C** Mutation analysis by T-cloning and Sanger sequencing in all piglets. WT sequence is shown at the top. PAM sites are highlighted in red; target sequences are shown in green. **D** The relative expression of *DMD* mRNA in DMD-delE51 pigs and the age-matched wild-type pigs. *DIA* diaphragm, *GAST* gastrocnemius; ***P < 0.001. **E** Western blotting showed the disrupted expression of dystrophin in DMD-delE51 pigs. **F** IHC analysis of dystrophin expression in heart, diaphragm and gastrocnemius muscles. Scale bars: 50 µm and 100 µm

Figure 2A shows the flowchart of the construction process of Bama miniature pigs with *DMD* exon 51 mutations (DMD-delE51 pigs). Large white pigs were selected as surrogate sows, and a total of 200 embryos were transferred to each surrogate. In total, three pregnant surrogates were carried to term, and 15 piglets were delivered (Additional file 1: Table S5). DNA gel electrophoresis and sequencing analysis of all piglets showed that 9 of the newborns carried mutations at the target locus (Fig. 2B, C). To examine whether the *DMD* mutations in pigs disrupt the expression of the *DMD* gene, we performed RT-PCR analysis of *DMD* gene expression. As shown in Fig. 2D, the expression of the *DMD* gene in the cardiac and skeletal muscle of DMD-delE51 pigs was significantly lower than that of WT pigs. To further examine the expression of dystrophin protein in the muscle of these pigs, we performed Western blotting and immunohistochemistry staining. Compared with WT controls, the expression of dystrophin protein in DMD-delE51 pigs was disrupted (Fig. 2E, F). These results demonstrated that the mutations in *DMD* exon 51 disrupted the expression of the *DMD* gene and dystrophin protein.

### Muscular dystrophy and cardiomyopathy presentation in DMD-delE51 pigs

**Fig. 3:**
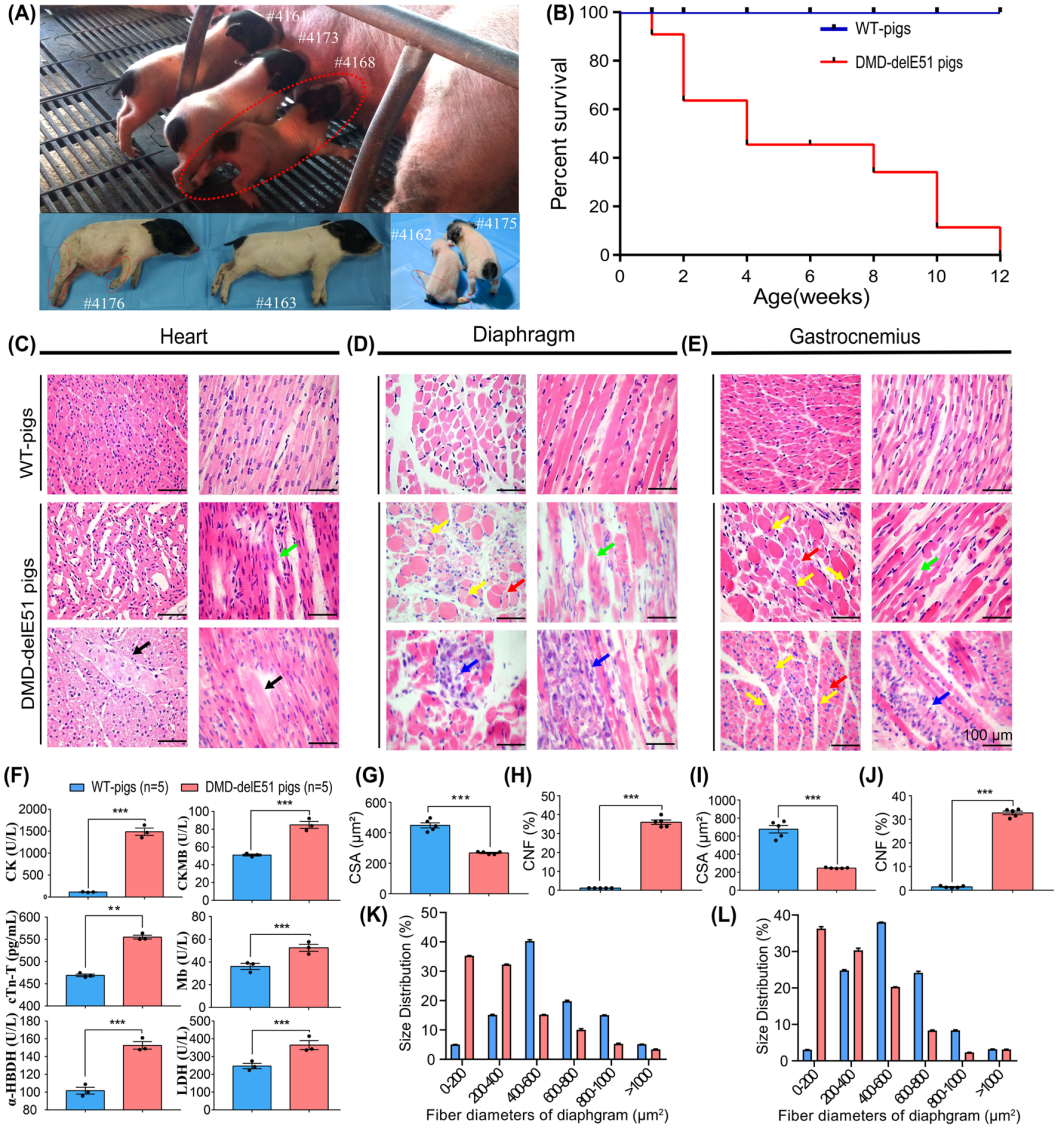
Development of muscular dystrophy and cardiomyopathy in DMD-delE51 pigs. **A** Piglets carrying *DMD* exon 51 mutations showed abnormal posture. **B** Survival curves of DMD-delE51 pigs and WT-pigs. **C**–**E** H&E staining of heart, diaphragm and gastrocnemius muscle sections from WT and DMD-delE51 pigs at the age of 12 weeks. Scale bars: 100 µm. Fiber fracture (green arrows) and hypertrophic fiber (black arrows) were seen in heart sections. Excessive fiber size variation (red arrows), central nucleated fibers (yellow arrows), fiber fracture (green arrows) and inflammatory cell infiltration (blue arrows) were readily visible in muscle sections. **F** Serum biochemical profiles of WT and DMD-delE51 pigs. ***P < 0.001, **P < 0.01 and *P < 0.05. **G**, **I** Quantification of cross-sectional area (CSA) of diaphragm and gastrocnemius muscle fibers in WT and DMD-delE51 pigs. **H**, **J** Quantification of centrally nucleated fiber (CNF) percentage in WT and DMD-delE51 pigs. **K**, **L** Size distribution of diaphragm and gastrocnemius muscle in WT and DMD-delE51 pigs. Scale bars: 100 µm. ***P < 0.001, **P < 0.01 and *P < 0.05; n = 5

As shown in Fig. 3A, the DMD-delE51 pigs exhibited obvious hindlimb paralysis compared with their WT littermates. The DMD-delE51 pigs began to die postnatally, with the mortality rate reaching 100% within 12 weeks, whereas WT piglets had a normal lifespan (Fig. 3B). To examine the histopathology of the DMD-delE51 pigs, we performed H&E staining of cardiac, diaphragm and gastrocnemius muscle sections from the pigs at 12 weeks of age. As shown in Fig. 3C, the cardiac muscle sections of DMD-delE51 pigs displayed irregularly arranged and cracked myocardial fibers, significantly widened fibrous gaps and hypertrophic myocardial fibers. Meanwhile, typical muscular dystrophy signs were observed in skeletal muscles, as evidenced by increased fiber size variation, centrally nucleated fibers and inflammatory cell infiltration (Fig. 3D, E). As shown in Fig. 3G, I, the average fiber area of the diaphragm and gastrocnemius muscles of DMD-delE51 pigs was significantly lower than that of WT pigs due to the cycles of degeneration and regeneration. In addition, a significantly increased percentage of muscle fibers with central nuclei was observed in DMD-delE51 pigs, as shown in Fig. 3H, J, and fiber size distribution revealed a significant increase in smaller fibers (Fig. 3K, L). Moreover, muscular dystrophy and cardiomyopathy biomarkers were founded elevated in the serum samples of DMD-delE51 pigs. As shown in Fig. 3F, DMD-delE51 pigs exhibited significantly elevated levels of serum creatine kinase (CK), creatine kinase MB isoenzyme (CKMB), cardiac troponin T (cTn-T), myoglobin (Mb), α-hydroxybutyrate dehydrogenase (α-HBDH) LDH compared with WT pigs (P < 0.001, ***; 0.001 < P < 0.01, **). Taken together, these results demonstrated that the DMD-delE51 pigs developed DMD and cardiomyopathy.

### Pathological alterations in the small intestine linked to malnutrition in DMD-delE51 pigs

**Fig. 4:**
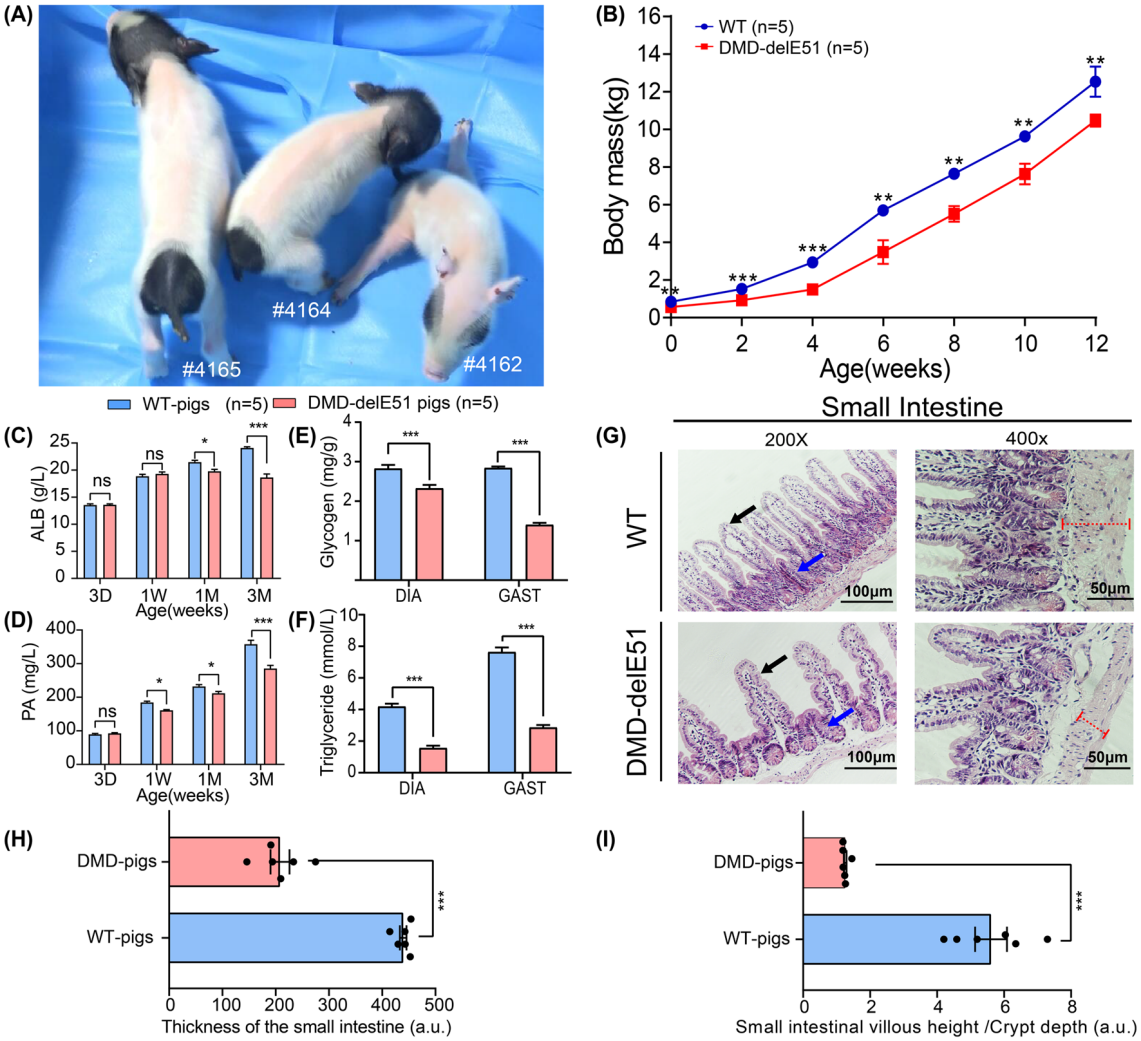
DMD-delE51 pigs exhibited symptoms of malnutrition. **A** Photograph of DMD-delE51 pigs and WT control at the age of 1 week. **B** Body mass of DMD-delE51 and WT pigs from birth to 12 weeks of age. **C**, **D** Serum ALB and PA levels of WT and DMD-delE51 pigs. **E**, **F** The contents of glycogen and triglyceride in diaphragm and gastrocnemius muscle. **G** H&E staining of the small intestine sections of WT and DMD-delE51 pigs. Red dotted lines indicate the thickness of the intestinal wall, black arrows indicate the height of the small intestine villi and blue arrows indicate the depth of the small intestine crypt. **H** The thickness of small intestinal wall in WT and DMD-delE51 pigs. **I** The relative ratio of small intestine villus height/crypt depth of WT and DMD-delE51 pigs. Scale bars: 50 µm and 100 µm. ***P < 0.001, **P < 0.01 and *P < 0.05; n = 5

The growth and development of DMD-delE51 pigs and WT pigs were monitored. Compared with their WT littermates, DMD-delE51 pigs exhibited smaller body size (Fig. 4A) and lighter body weight (Fig. 4B, ***P < 0.001, **P < 0.01). Serological tests showed that the levels of serum albumin (ALB) and prealbumin (PA) were significantly reduced in DMD-delE51 pigs, indicating malnutrition (Fig. 4C, D). Indeed, measurement of muscle glycogen and triglyceride supported that malnutrition occurred in DMD-delE51 pigs (Fig. 4E, F, ***P < 0.001, *P < 0.05).

To examine the histopathology of the GI tract in DMD-delE51 pigs, we performed H&E staining of small intestinal tissue sections from the pigs at 12 weeks of age. As shown in Fig. 4G, I and Additional file 1: Fig. S3A, the intestinal villus height of DMD-delE51 pigs was significantly decreased compared with that of the controls (***P < 0.001), and the ratio of villus height to crypt depth was approximately 60% reduced. Meanwhile, the thickness of the intestinal wall was reduced by approximately 40% (Fig. 4H, ***P < 0.001). In addition, we also examined the expression of the *DMD* gene and dystrophin protein in the small intestine. The expression of the *DMD* gene and dystrophin protein were significantly disrupted in the stomach and small intestine (Additional file 1: Fig. S3B–E, ***P < 0.001).

### DMD-delE51 pigs suffered from gastric dysfunction

**Fig. 5:**
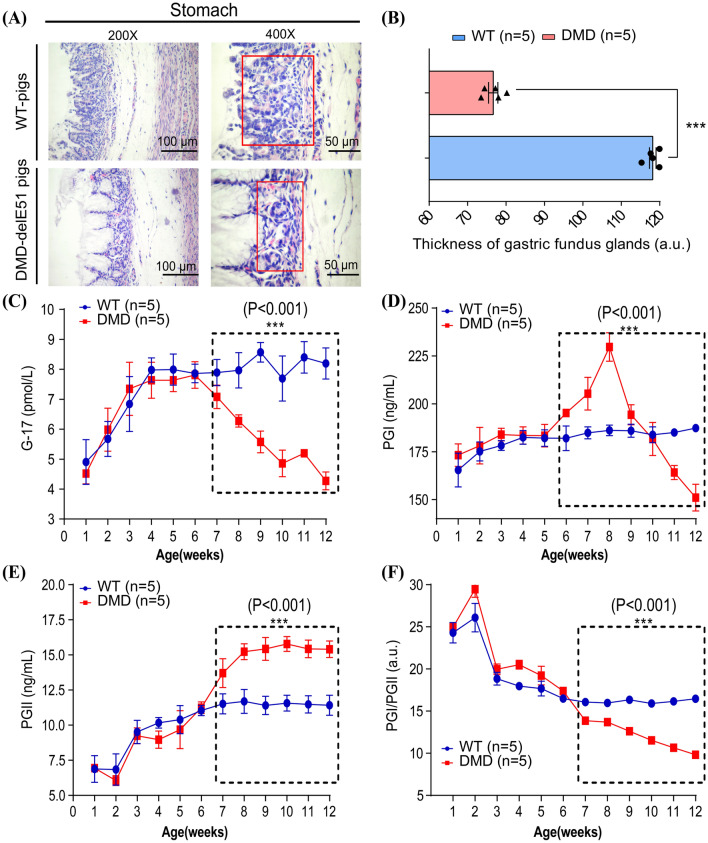
DMD-delE51 pigs suffered from atrophic gastritis. **A** H&E staining of stomach sections from WT and DMD-delE51 pigs at the age of 12 weeks. The thickness of gastric fundus glands (red rectangle) was reduced in DMD-delE51 pigs. **B** Quantification of the thickness of gastric fundus glands in WT and DMD-delE51 pigs. **C** Measurements of serum G-17 in WT and DMD-delE51 pigs. **D** Measurement of serum PGI in WT and DMD-delE51 pigs. **E** Measurement of serum PGII in WT and DMD-delE51 pigs. **F** The ratio of PGI/PGII in WT and DMD-delE51 pigs. Scale bars: 50 µm and 100 µm. ***P < 0.001, **P < 0.01 and *P < 0.05; n = 5

H&E staining of the stomach revealed that the gastric gland of DMD-delE51 pigs was significantly thinner than that of WT pigs (Fig. 5A, B, ***P < 0.001). Importantly, compared with WT pigs, serum gastrin 17 (G-17) levels in DMD-delE51 pigs were significantly downregulated at week 7 (Fig. 5C, ***P < 0.001), serum pepsinogen I (PGI) levels were upregulated at week 6 and reached the peak at week 8, but then sharply declined to a minimum by week 12 (Fig. 5D, ***P < 0.001). Serum pepsinogen II (PGII) levels began to be significantly upregulated at week 7 and remained higher than those in WT pigs (Fig. 5E, ***P < 0.001). The ratio of PGI/PGII (PGR) at week 7 was significantly lower in DMD-delE51 pigs than in WT pigs (Fig. 5F, ***P < 0.001). Collectively, DMD-delE51 pigs with disrupted dystrophin expression exhibited morphological abnormality and functional impairment in the stomach.

## Discussion

In this study, we established a new pig model of DMD using CRISPR-genome editing and SCNT, and studied the pathological alterations in the gastrointestinal tract. We demonstrated that the DMD-delE51 pigs shown these typical DMD phenotypes: dyskinesia, muscular dystrophy and cardiomyopathy. More importantly, we have found significant pathological alterations in the gastrointestinal tract of DMD-delE51 pigs. In DMD-delE51 pigs, the intestinal villus height, the ratio of villus height to crypt depth, and the thickness of the intestinal wall and gastric gland decreased significantly. In addition, the levels of ALB, PA, glycogen and triglyceride were declined significantly. Furthermore, the ratio of PGI/PGII was significantly lower in DMD-delE51 pigs than in WT pigs.

Previous research has demonstrated that the gastric gland and intestinal wall are the most important parts of the digestive system [25]. The thickness of the fundic gland and intestinal wall is closely related to the rhythmic contraction of the gastrointestinal tract and mechanical digestion, which reflects the rates of digestion and absorption of nutrients. In addition, the ratio of villus height to crypt depth comprehensively reflects the digestive and absorption function of the small intestine [26]. When the ratio decreases, the mucous membrane is likely to be damaged, and the digestive and absorptive capacity is reduced, often accompanied by diarrhea and growth inhibition [27]. In this study, DMD-delE51 pigs showed decreased gastric fundic gland and intestinal wall thickness, shortened intestinal villus height, reduced levels of ALB, PA, glycogen and triglyceride, similar to clinical manifestations of malnutrition in human patients [28]. Previous research has shown abnormal levels of G-17, PGI, PGII and PGR in serum samples of patients with gastritis [29]. In atrophic gastritis (AG) dominated by antral atrophy, antral mucosal atrophy can lead to a decrease in the number of G cells and a decrease in the secretion of G-17, resulting in a decrease in the content of G-17 in blood circulation [30]. Moreover, the low levels of serum PGI and PGR are biological markers of gastric body atrophy, and the level of serum PGI decreases gradually with increasing severity of mucosal atrophy [31]. In DMD-delE51 pigs, the changes of G-17, PGI and PGII were highly similar to those human patients with AG [29].

Atrophic gastritis was the final consequence of an inflammatory process that ultimately leads to loss of appropriate mucosal glands [32]. After suffering from atrophic gastritis, patients were often prone to severe gastrointestinal discomfort, unable to eat normally, and thus aggravated the symptoms of malnutrition [33]. Malnutrition may be due to reduced food intake and/or increased energy expenditure. Studies have shown that malnutrition and weight loss are likely to accelerate disease progression, increase morbidity and reduce survival. Therefore, we speculated that dystrophin protein deficiency caused changes in the morphology and function of the gastrointestinal tract, thereby affecting the digestion and absorption of nutrients, leading to malnutrition in DMD-delE51 pigs and aggravating the disease progression of DMD and premature death of individuals.

## Conclusion

Taken together, the pathological alterations in the GI tract are of clinical importance and likely contribute to the overall disease progression via malnutrition. These findings highlight the need to consider the gastrointestinal dysfunction as an additional therapeutic target for DMD patients.

## Materials and methods

### Ethics statement

All animal studies were approved by the Animal Welfare and Research Ethics Committee at Jilin University, and all procedures were conducted strictly in accordance with the Guide for the Care and Use of Laboratory Animals. All surgeries were performed under anesthesia, and every effort was made to minimize animal suffering (SY201901013).

### Construction of Cas9/sgRNA targeting vector

The CRISPR/Cas9 system was constructed as previously described [34]. Briefly, the plasmid containing the U6-sgRNA and Cas9 expression elements was obtained from Addgene (#42230). The targeting sgRNA is CTTGGACAGAACTTACCGAC. A pair of complementary sgRNA oligo DNAs were synthesized, annealed into double-strand DNAs, and ligated to the *BbsI* sites of the vector to form the intact plasmid, which was confirmed by sequence analysis.

### Isolation and culture of PFFs

The isolation and culture of PFFs were performed as previously described [35]. Thirty-three-day-old fetuses were chosen and separated from Bama miniature sows. First, fetuses, without the head, tail, limb bones, and viscera, were cut into small pieces. Then, these small pieces were digested and cultured in DMEM (GIBCO) supplemented with 15% fetal bovine serum (FBS) at 39 °C and 5% CO_2_ in a humidified incubator. PFFs at passage 1 were frozen in FBS containing 10% dimethyl sulfoxide (DMSO).

### Electrotransfection and single-cell colony selection

First, PFFs were thawed and cultured in 10-cm culture dishes. Then, 3 × 10^6^ PFFs were electrotransfected with 200 μL of Opti-MEM (GIBCO) using 2-mm gap cuvettes and a BTX ECM 2001 electroporator. The parameters for electrotransfection were as follows: 340 V, 1 ms, 3 pulses for 1 repeat. During these experiments, a total of 30 μg plasmids were added to the reaction media. At 36 h after electrotransfection, the cells were plated into ten 10-cm dishes at a density of 4 × 10^3^ cells per dish. Single-cell colonies were picked and cultured in 24-well plates. When the plates reached 90% confluence, 10% of cells from each plate was lysed using 10 μL of lysis buffer (0.45% NP-40 plus 0.6% Proteinase K) for 60 min at 56 °C and then 10 min at 95 °C. The lysate was used as a template for PCR. The forward and reverse primers were 5ʹ-CAGCTAAACAGAGTAAAGAG-3ʹ and 5ʹ-GATTTCCCTAGAGTCCACTT-3ʹ, respectively. The PCR conditions were 94 °C for 5 min; 94 °C for 30 s, 55 °C for 30 s, and 72 °C for 1 min for 35 cycles; 72 °C for 5 min; and hold at 16 °C. The PCR products were sequenced, and some PCR products were ligated into the PLB vector (Tiangen, Beijing, China) and sequenced to identify the mutations. The positive cell colonies were expanded and cryopreserved.

### Off-target assay

Potential off-target sites (OTSs) were predicted by scanning the porcine genome using BLAST based on the homology to the sgRNA plus protospacer adjacent motif (PAM). The genomic DNA of the mutant cell clones was analyzed via PCR and DNA sequencing to determine the target effects. The primer sequences used for analyzed the off-target activities are listed in Additional file 1: Table S2.

### Somatic nuclear transfer technology (SCNT) and genotyping of mutant piglets

Somatic cell nuclear transfer and embryo transfer were performed as previously reported [36]. Positive colonies were screened and selected as donor cells for SCNT. First, a single donor cell was microinjected into the enucleated pig oocyte. Second, reconstructed embryos were electroactivated. Finally, embryos were transferred into synchronized recipient pigs. After piglets were delivered, genomic DNA samples were extracted from the tail tissue for genotyping.

### Survival curve and bodyweight

The bodyweights of age- and sex-matched WT and *DMD*-modified pigs were measured biweekly. A minimum of three individual animals of *DMD*-modified pigs was used in all experiments.

### Serum biochemical analysis

After piglets were born, serum samples were collected and measured by enzyme-linked immunosorbent assay (ELISA) following the manufacturer’s instructions, in an infinite 200 PRO Microplate Reader (Tecan, Switzerland). Samples were measured in triplicate, and the absorbance was monitored at 37 °C.

### Neutral red dye (NRD) uptake assay

The cell viability was analyzed by the Neutral Red Cell Proliferation and Cytotoxicology Assay Kit (Beyotime, China). The absorbance (OD) was measured at 540 nm, and the value at the reference wavelength of 630 nm was subtracted. The assay was performed on each cell clone in triplicate, and values were averaged from 4 to 6 wells per plate.

### Lactate dehydrogenase (LDH) assay

LDH activity in the medium was measured using the LDH assay kit (Beyotime, China) according to the manufacturer’s instruction. The OD was measured by an infinite 200 PRO Microplate Reader (Tecan, Switzerland) at a wavelength of 490 nm. LDH activity was calculated according to the formula provided by the instruction.

### The measurement of muscle glycogen and triglyceride

Muscle glycogen and triglyceride contents were measured using the Liver/Muscle glycogen assay kit and Triglyceride assay kit (Nanjing Jiancheng Bioengineering Institute, China) according to the manufacturer’s instruction. The absorbance was measured by an infinite 200 PRO Microplate Reader (Tecan, Switzerland). Samples were measured in triplicate at 37 °C.

### Analysis of apoptotic cells by flow cytometry

Annexin V-FITC antibody immunofluorescence combined with propidium iodide (PI) was used to perform a fluorescent analysis of apoptosis, according to the instructions of the Annexin V-FITC apoptosis detection kit (Beyotime, China). A total of 1 × 10^5^ cells were collected and incubated with Annexin V-FITC and PI in the provided binding buffer for 25 min in the dark at 4 °C, and analyzed by flow cytometry.

### Western blotting

Equal amounts of proteins were separated through SDS-PAGE on a 5% separating gel, and the protein bands were electrophoretically transferred to polyvinylidene fluoride (PVDF) membranes. Then, the membranes were blocked for 2 h in TBST buffer with 5% milk at room temperature. The membranes were subsequently incubated with the primary antibodies (rabbit anti-light chain-3B (LC3B) antibody, BM4827, Boster, 1:400; anti-dystrophin antibody, ab15277, Abcam, 1:400) overnight at 4 °C. After wash 3 times for 10 min with TBST buffer, the membranes were incubated for 1 h with the secondary antibody. Finally, the protein bands were detected with the ECL-Plus Western blotting reagent.

### Quantitative reverse transcription PCR (RT-PCR)

For detection of relative mRNA expression of the *DMD* gene, total RNA was isolated from muscle samples using TRNzol-A^+^ Reagent (TIANGEN, DP421) following the manufacturer’s recommendations. The RNA (1 μg) was reverse-transcribed (RT) to generate cDNA using a FastKing RT Kit (with gDNase) (TIANGEN, KR116) according to the manufacturer’s manual. The reaction conditions were 95 °C for 5 min and 10 s; 60 °C for 20 s and 72 °C for 30 s for 40 cycles; and 95–55 °C for 30 s (melting curve). The fluorescence intensity and amplification plots were analyzed by a BIO-RAD iCycler Thermal Cycler with iQ5 Optical Module for RT-PCR (Bio-Rad, ABI 7500, iQ5). The results were expressed via the comparative cycle threshold (CT) method as described before, and expression levels were represented by fold changes over values derived from healthy pigs. *GAPDH* was utilized as a reference gene. The primers used in RT-PCR are listed in Additional file 1: Table S6.

### Immunohistochemistry (IHC)

Dystrophin expression and location were examined in fixed sections. Muscle samples were fixed in 4% paraformaldehyde (PFA), embedded in paraffin, and sectioned at 5 μm after harvest. IHC was performed according to standard techniques. Briefly, muscle sections were deparaffinized, and antigen retrieval was performed. After slices were cooled to 25 °C and washed twice with PBS, the slides were blocked with 5% BSA for 15 min at 25 °C. Then, the slides were incubated with the primary anti-dystrophin antibody (MANDYS8, GeneTex, 1:400) overnight at 4 °C. Following incubation and 3 washes with 0.05% Tween-20 in PBS, sections were incubated with Alexa Fluor 488-conjugated Affinipure goat anti-mouse IgG (H+L) (SA0006-1, ProteinTech, 1:100) for 1 h at 25 °C and washed again. Slides were mounted with SlowFade Gold Antifade Reagent with DAPI (Invitrogen). For analysis of protein abundance following IHC, 5 nonoverlapping pictures were randomly taken from each section.

### Hematoxylin and eosin (H&E) staining

Fresh tissues including heart, gastrocnemius, diaphragm, stomach and intestine, were fixed in 4% PFA, embedded in paraffin, and sectioned at 5 μm. H&E staining was performed with standard techniques. Briefly, sections were incubated in Mayer’s hematoxylin. Then, sections were rinsed with tap water and counterstained with 1% eosin. Finally, the sections were dehydrated, and coverslips were applied. Five nonoverlapping pictures were randomly taken from each muscle section.

### Morphometric analysis of muscle

Morphometric analyses were performed on H&E-stained muscle sections of *DMD* exon 51-modified pigs and age-matched WT controls following the manufacturer’s instructions, and five different regions were counted per section. The fiber size and percentage of central nucleated fibers were calculated by ImagePro Plus software (v6.0, Media Cybernetics, Silver Spring, MD, USA).

### Statistical analysis

All data are expressed as the means ± standard error of the mean (SEM). Statistical differences were determined by unpaired Student’s t-test for two group comparisons and one-way ANOVA with Bonferroni’s post-tests for multiple group comparisons using GraphPad Prism 7.0 software.

## Supplementary Information

**Additional file 1: Table S1.** List of putative off-target sites (PAM sequences are labeled in blue. Base substitutions are shown in red.). **Table S2.** List of primers for PCR amplification of off-target sites. **Table S3.** Identification and classification of PFFs carrying different mutations in DMD exon 51. **Table S4.** Analysis of blastocyst development rate of DMD exon 51-modified PFFs. **Table S5.** SCNT results for the generation of DMD-delE51 pigs. **Table S6.** List of primers used for RT-PCR. **Figure S1.** Sequence analysis of pig dystrophin and design of CRISPR-targeting strategy. **Figure S2.** Analysis of PFFs with DMD mutations. **Figure S3.** Dystrophin expression in porcine stomach and small intestine.

## Data Availability

All relevant data supporting the key findings of this study are available within the article and its Additional Information files or from the corresponding author upon reasonable request.

## Abbreviations

AG: Atrophic gastritis
ALB: Albumin
α-HBDH: α-Hydroxybutyrate dehydrogenase
CK: Creatine kinase
CKMB: Creatine kinase MB isoenzyme
cTn-T: Cardiac troponin T
DMD: Duchenne muscular dystrophy
GI: Gastrointestinal
LDH: Lactate dehydrogenase
Mb: Myoglobin
NRD: Neutral red dye
PA: Prealbumin
PFFs: Porcine fetal fibroblasts
PGI: Pepsinogen I
PGII: Pepsinogen II
PGR: Ratio of PGI/PGII
SCNT: Somatic nuclear transfer technology
sgRNA: Single guide RNA
WT: Wild-type
