# Supplementary material for: Pathological alterations in the gastrointestinal tract of a porcine model of DMD
Source: Cell Biosci. 2021 Jul 15;11:131. doi: 10.1186/s13578-021-00647-9 (PMC8281460; doi:10.1186/s13578-021-00647-9)
Supplement: Supplementary file 1 — Additional file 1: Table S1. List of putative off-target sites (PAM sequences are labeled in blue. Base substitutions are shown in red.). Table S2. List of primers for PCR amplification of off-target sites. Table S3. Identification and classification of PFFs carrying different mutations in DMD exon 51. Table S4. Analysis of blastocyst development rate of DMD exon 51-modified PFFs. Table S5. SCNT results for the generation of DMD-delE51 pigs. Table S6. List of primers used for RT-PCR. Figure S1. Sequence analysis of pig dystrophin and design of CRISPR-targeting strategy. Figure S2. Analysis of PFFs with DMD mutations. Figure S3. Dystrophin expression in porcine stomach and small intestine. [file 13578_2021_647_MOESM1_ESM.docx]

**Supplementary Information**

**Table S1**: List of putative off-target sites (PAM sequences are labeled in blue. Base substitutions are shown in red.)

| **sgRNA** | CTTGGACAGAACTTACCGACTGG |
| --- | --- |
| **OTS1** | CCAGTACAGAACTTACAGACCGG |
| **OTS2** | CTTTGATAGGACTTACCGATTAG |
| **OTS3** | CTTCTCCAGAACTTACCGCCCAG |
| **OTS4** | CTTGTACAGAAATCACCGACCAG |
| **OTS5** | CTTGGACACATCTTACCAACAGG |
| **OTS6** | CCTGTACAGAACTTACCTATGAG |
| **OTS7** | CTTGGCCAGCATTTACCGTCAGG |
| **OTS8** | ATTTGACAGAACATACCAACAGG |

**Table S2**: List of primers for PCR amplification of off-target sites.

| **Primers** | **Sequences (5' to 3')** | **Amplicon (bp)** |
| --- | --- | --- |
| DMD- sgRNA-OTS1 | AAGGAAATTGAGACTCAGAGAAGA | 502 |
|  | TGCTTTTCATTGGCTCTGGC |  |
| DMD- sgRNA-OTS2 | TGCCAGTGTGGTTGGTTTCT | 551 |
|  | CCAGTCCATTCCCCCATCAC |  |
| DMD - sgRNA-OTS3 | GTTTACCGCAGACCCACAGA | 589 |
|  | GTGCGTAGAGACCCAAACCA |  |
| DMD - sgRNA-OTS4 | GGCTGGTCATGGTTAGCACT | 522 |
|  | CTGAACACCCTTCCTCCACC |  |
| DMD - sgRNA-OTS5 | TTTGACCCCAATCCATGCGT | 577 |
|  | TGCTCTATGCCACTTCGCTT |  |
| DMD - sgRNA-OTS6 | TGTGTCTTGGTGGGTGATGG | 508 |
|  | GTGTGGATGGGTGTATGCCA |  |
| DMD - sgRNA-OTS7 | AGGGGCTTATGCTTGTGGTC | 522 |
|  | TCAGAAGCCTGCCCTTCATG |  |
| DMD - sgRNA-OTS8 | GGTCCTGACCCTTTGGATGT | 593 |
|  | AGGCTGAATTATCTGAGTGCCA |  |

**Table S3**: Identification and classification of PFFs carrying different mutations in *DMD* exon 51.

| **Types of clone cell** | **Numbers** | **Percentage (%)** |
| --- | --- | --- |
| Wild type (WT) | 290 | 72.50 |
| DMD-KO-Type 1 (T1) | 10 | 2.50 |
| DMD-KO-Type 2 (T2) | 8 | 2.00 |
| DMD-KO-Type 3 (T3) | 10 | 2.50 |
| DMD-KO-Type 4 (T4) | 15 | 3.75 |
| DMD-KO-Type 5 (T5) | 23 | 5.75 |
| DMD-KO-Type 6 (T6) | 18 | 4.50 |
| DMD-KO-Type 7 (T7) | 26 | 6.50 |
| In total | 400 | 100.00 |

**Table S4**: Analysis of blastocyst development rate of DMD exon 51-modified PFFs.

| **Cell Clone Types** | **SCNT Repeats** | **Nuclear Cell Clones**  **Number** | **Blastocyst Number** | **Blastocyst Development**  **Rate（%）** |
| --- | --- | --- | --- | --- |
| T1 | 1 | 80 | 12 | 15.00 |
|  | 2 | 80 | 14 | 17.50 |
|  | 3 | 80 | 13 | 16.30 |
| T2 | 1 | 80 | 14 | 17.50 |
|  | 2 | 80 | 14 | 17.50 |
|  | 3 | 80 | 13 | 16.30 |
| T3 | 1 | 80 | 12 | 15.00 |
|  | 2 | 80 | 18 | 22.50 |
|  | 3 | 80 | 13 | 16.30 |
| T4 | 1 | 80 | 13 | 16.30 |
|  | 2 | 80 | 12 | 15.00 |
|  | 3 | 80 | 18 | 22.50 |
| T5 | 1 | 80 | 15 | 18.80 |
|  | 2 | 80 | 13 | 16.30 |
|  | 3 | 80 | 12 | 15.00 |
| T6 | 1 | 80 | 15 | 18.80 |
|  | 2 | 80 | 14 | 17.50 |
|  | 3 | 80 | 15 | 18.80 |
| T7 | 1 | 80 | 14 | 17.50 |
|  | 2 | 80 | 15 | 18.80 |
|  | 3 | 80 | 15 | 18.80 |
| WT | 1 | 80 | 17 | 21.30 |
|  | 2 | 80 | 17 | 21.30 |
|  | 3 | 80 | 14 | 17.50 |

**Table S5**: SCNT results for the generation of DMD-delE51 pigs.

| **Target gene** | **Transferred embryos** | **No. recipients** | **No.(%) pregnancies** | **No. born** | **No. (%) mutants** |
| --- | --- | --- | --- | --- | --- |
| *DMD* | 200 | 1 | 1 | 4 | 2 |
|  | 200 | 1 | 0 | 0 | 0 |
|  | 200 | 1 | 1 | 4 | 3 |
|  | 200 | 1 | 0 | 0 | 0 |
|  | 200 | 1 | 1 | 7 | 4 |
| Total | 1000 | 5 | 3 | 15 | 9 |

**Table S6**: List of primers used for RT-PCR.

| RT-*DMD*-F (5’-3’) | CCCTGGACTGACCACTAT |
| --- | --- |
| RT-*DMD*-R (5’-3’) | CTCTGTGATTTTATAACTCG |
| RT-*GAPDH*-F (5’-3’) | ATCCTGGGCTACACTGAGGA |
| RT-*GAPDH*-R (5’-3’) | TGTCGTACCAGGAAATGAGCT |


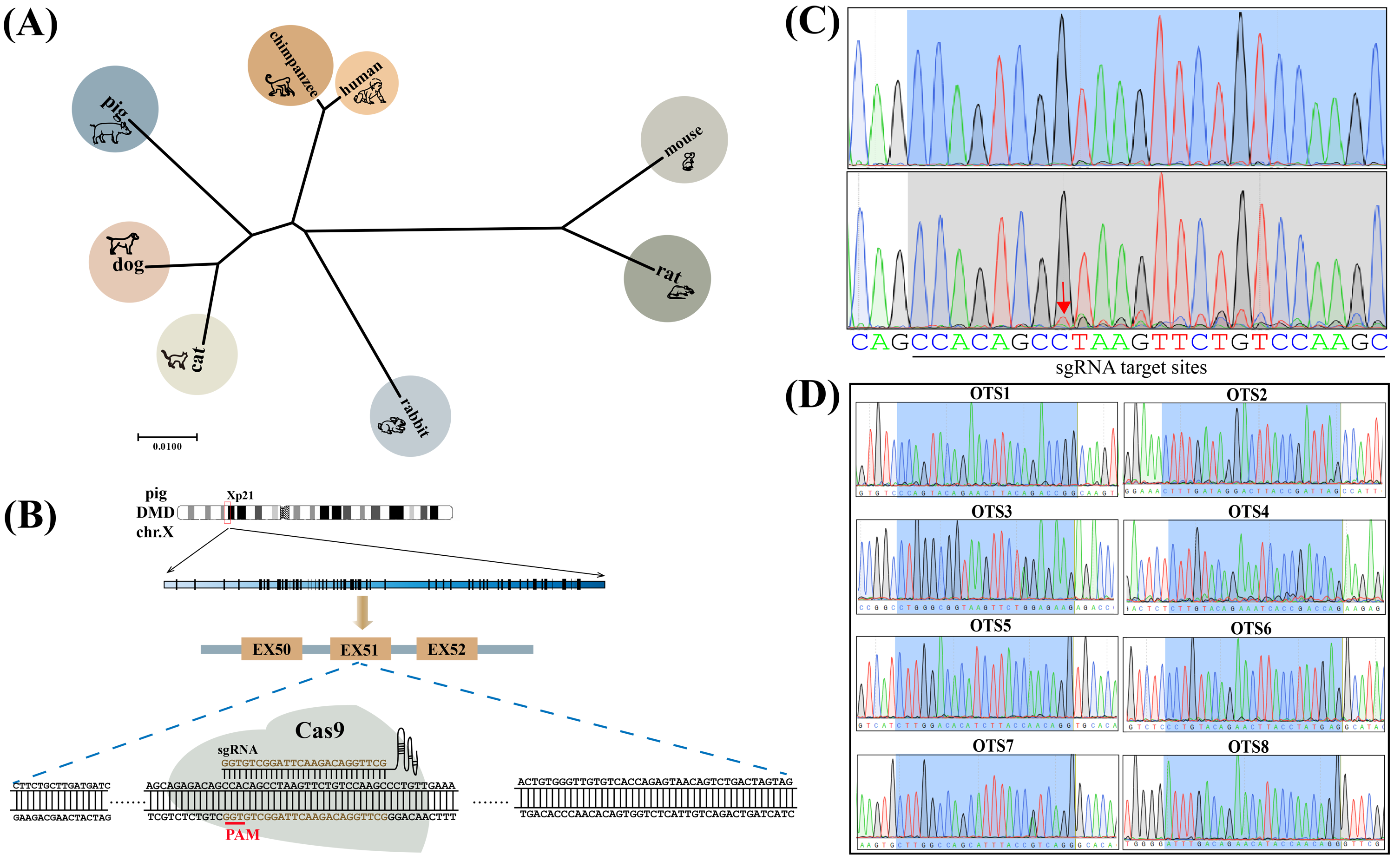


**Figure S1**. **Sequence analysis of pig dystrophin and design of CRISPR-targeting strategy**. (A) Comparison of amino acid sequences of dystrophin proteins among different species. (B)Schematic representation of the sgRNA targeting porcine *DMD* exon 51. PAM is highlighted in red. (C) Sanger sequencing traces of PCR amplicons from WT and electrotransfected PFFs. The cleavage sites are labeled with a red arrow. (D) The analysis of off-target sites (OTS). The corresponding sequencing chromatograms for the top OTS are shown.


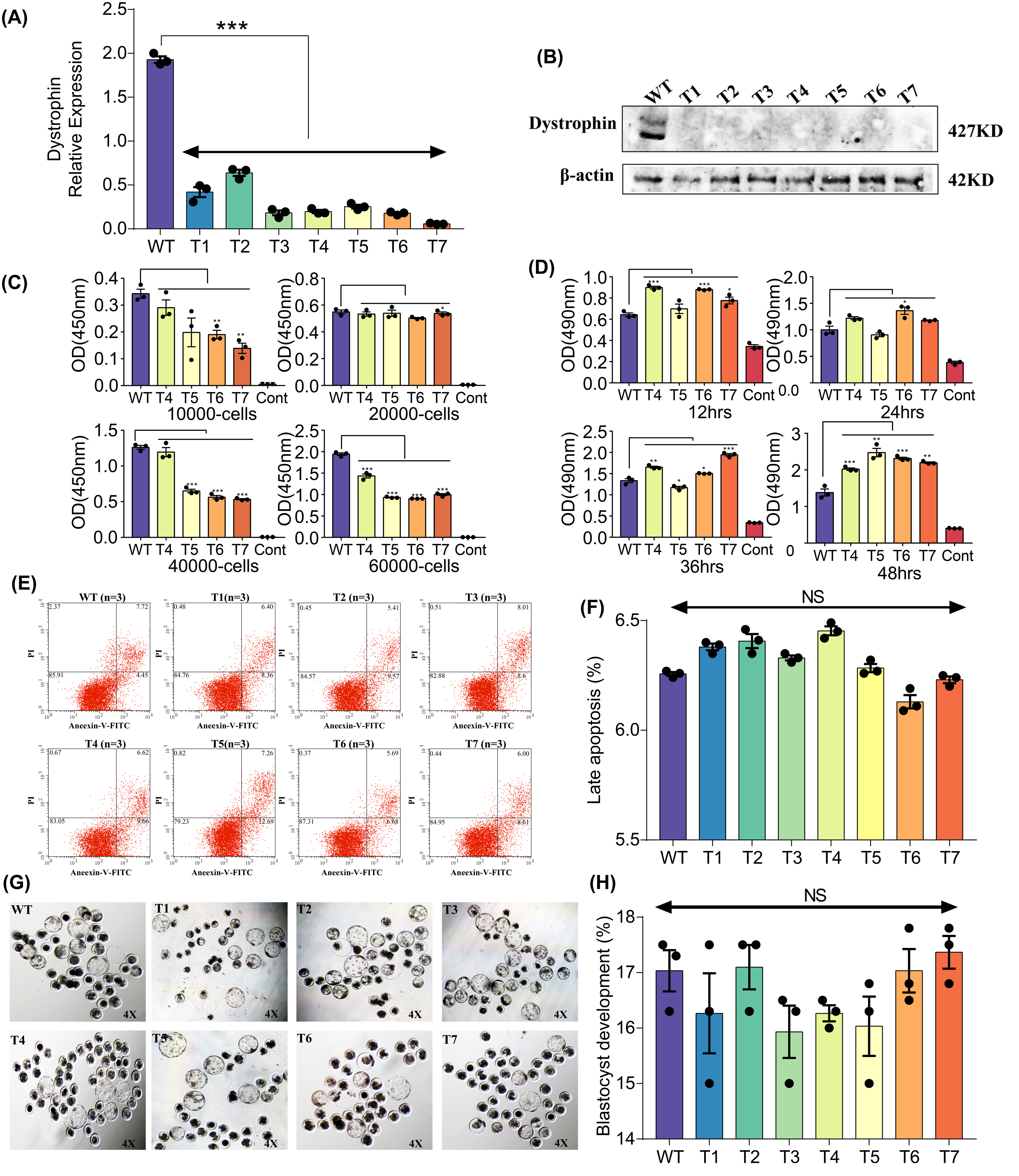


**Figure S2. Analysis of PFFs with *DMD* mutations.** The relative expression of *DMD* mRNA in DMD-edited and WT PFFs. ***P< 0.001. (B) Western blotting analysis of dystrophin expression in DMD-modified and WT PFFs. (C) NRD uptake analysis of PFF cell clones carrying DMD exon 51 mutations at different cell densities; ***P< 0.001, **P<0.01 and *P<0.05. (D) LDH activity in culture medium at different time periods. ***P< 0.001, **P<0.01 and *P<0.05. (E, F) Cell apoptosis analyzed by flow cytometry. (G) Representative images of blastocysts at 8.5 days after nuclear transfer. PFFs with DMD exon 51 modified could develop normally into blastocysts. (H) The analysis of blastocyst development rate of PFFs carrying DMD exon 51mutations. NS, no significant.


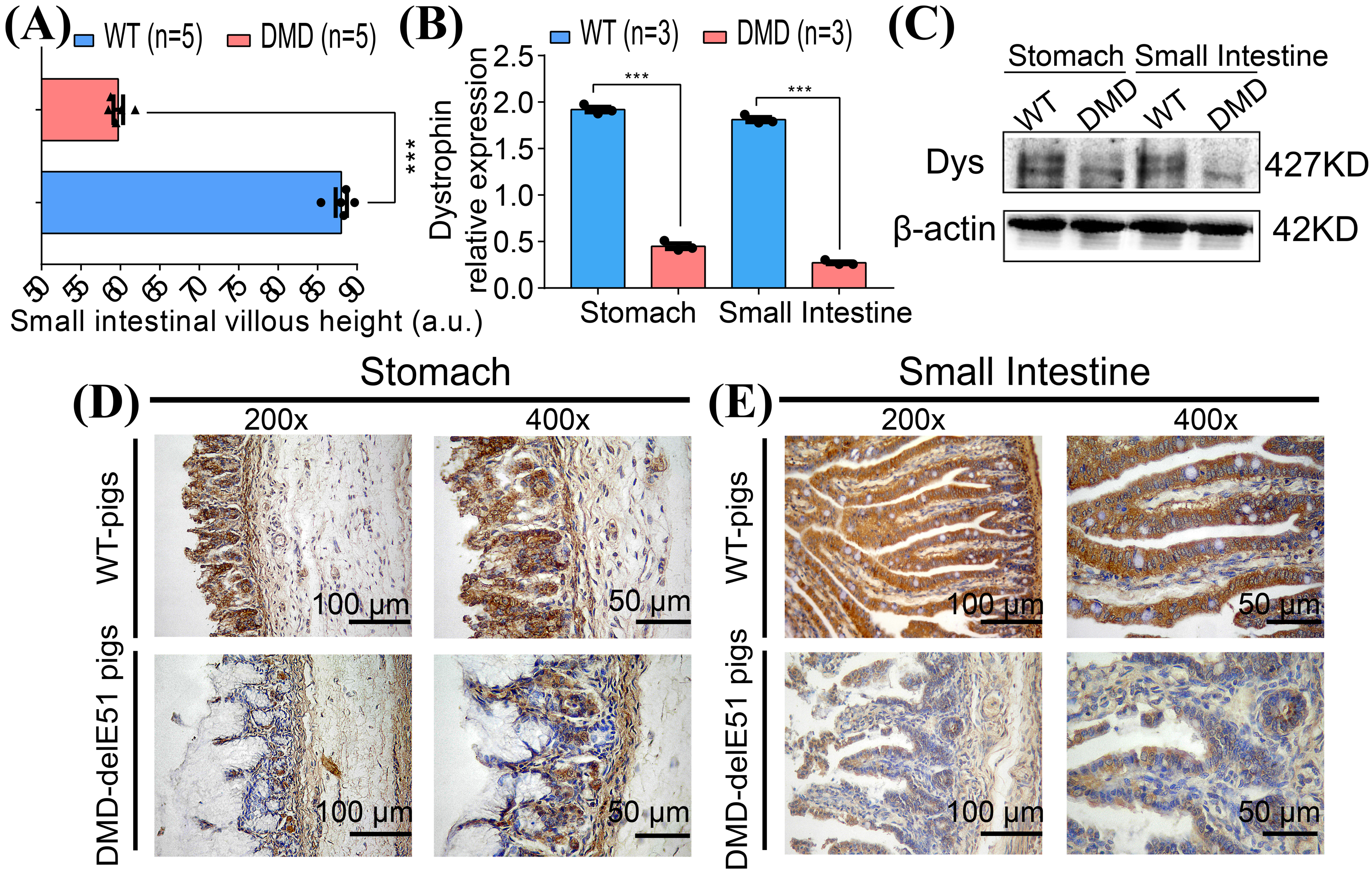


**Figure S3. Dystrophin expression in porcine stomach and small intestine.** (A) The relative height of small intestine villus in WT and DMD-delE51 pigs. *** p < 0.01. (B) The relative expression of *DMD* mRNA in stomach and small intestine from DMD-delE51 pigs and the age-matched wild-type pigs; ***P< 0.001. (C) Western blotting analysis of dystrophin (Dys) in stomach and small intestine of WT and DMD-delE51 pigs. (D, E) IHC staining of dystrophin in stomach (D) and small intestine (E) of pigs. Scale bars: 50 µm and 100 µm.
